# Challenges and promise of targeting miRNA in rheumatic diseases: a computational approach to identify miRNA association with cell types, cytokines, and disease mechanisms

**DOI:** 10.3389/fimmu.2023.1322806

**Published:** 2024-01-09

**Authors:** Farheen S. Shaikh, Ruby J. Siegel, Aayush Srivastava, David A. Fox, Salahuddin Ahmed

**Affiliations:** ^1^ Department of Pharmaceutical Sciences, Washington State University College of Pharmacy and Pharmaceutical Sciences, Spokane, WA, United States; ^2^ Department of Computer and Information Science and Engineering, Herbert Wertheim College of Engineering, University of Florida, Gainesville, FL, United States; ^3^ Department of Medicine, Division of Rheumatology and Clinical Autoimmunity Center of Excellence, University of Michigan Medical System, Ann Arbor, MI, United States; ^4^ Division of Rheumatology, University of Washington School of Medicine, Seattle, WA, United States

**Keywords:** miRNA, rheumatic diseases, epigenetics, therapeutics, personalized medicine

## Abstract

MicroRNAs (miRNAs) are small non-coding RNAs that alter the expression of target genes at the post-transcriptional level, influencing diverse outcomes in metabolism, cell differentiation, proliferation, cell survival, and cell death. Dysregulated miRNA expression is implicated in various rheumatic conditions, including ankylosing spondylitis (AS), gout, juvenile idiopathic arthritis (JIA), osteoarthritis (OA), psoriatic arthritis, rheumatoid arthritis (RA), Sjogren’s syndrome, systemic lupus erythematosus (SLE) and systemic sclerosis. For this review, we used an open-source programming language- PowerShell, to scan the massive number of existing primary research publications on PubMed on miRNAs in these nine diseases to identify and count unique co-occurrences of individual miRNAs and the disease name. These counts were used to rank the top seven most relevant immuno-miRs based on their research volume in each rheumatic disease. Individual miRNAs were also screened for publication with the names of immune cells, cytokines, and pathological processes involved in rheumatic diseases. These occurrences were tabulated into matrices to identify hotspots for research relevance. Based on this information, we summarize the basic and clinical findings for the top three miRNAs — miR-146, miR-155, and miR-21 — whose relevance spans across multiple rheumatic diseases. Furthermore, we highlight some unique miRNAs for each disease and why some rheumatic conditions lack research in this emerging epigenetics field. With the overwhelming number of publications on miRNAs in rheumatic diseases, this review serves as a ‘relevance finder’ to guide researchers in selecting miRNAs based on the compiled existing knowledge of their involvement in disease pathogenesis. This approach applies to other disease contexts with the end goal of developing miRNA-based therapeutics.

## miRNA: emerging importance and potential target in epigenetic regulation

Micro-RNAs (miRNA)s were an accidental discovery in *Caenorhabditis elegans* in 1993 in the Victor R. Ambros laboratory. The group observed that the altered expression of the gene *lin-4* could influence the LIN-14 protein levels despite steady *lin-14* transcription. The authors wondered why the *lin4* gene did not encode any protein and why the RNA products of *lin-4* had antisense complementarity to multiple sites in the 3’ untranslated region (UTR) of the *lin14* mRNA (1). This interesting observation led to the understanding that *lin4* regulated the expression of *lin-14* at the post-transcriptional level through an anti-sense RNA-RNA interaction. Further studies behind that interaction and regulation opened research avenues, ushering in a new era of ‘regulatory RNA’. As a result, what was initially discovered as “small temporal RNA” was later established and termed “microRNA” in 2011.

miRNAs are small single-stranded non-coding RNAs (~19-23-nucleotide length) that interfere with target protein expression. Since their discovery, hundreds of miRNAs have been identified and further classified on the basis of their impact on development, physiological processes, and diseases (2, 3). Since miRNA amounts in a cell can change rapidly, their altered expression is indicative of an early response and resulting cellular events initiated by external stimuli (4). miRNAs regulate cellular homeostasis by serving as nodes of signaling networks (5). Culminating evidence from research illustrated that even a single miRNA can regulate multiple (>200) genes, and conversely, multiple miRNAs can regulate a single gene (6, 7). This dynamic yet highly complex interaction of miRNAs and their target genes and how it is impacted by the abundance of miRNAs and the affinity and access to target mRNAs make miRNA a hot topic in biomedical research.

Concomitant to genetic polymorphism, epigenetic regulation plays an important role in the onset and progression of chronic inflammatory diseases, including rheumatic conditions. In addition to the well-established role of DNA methylation and histone acetylation, miRNAs and other non-coding RNAs have joined the league of known epigenetic regulators. These epigenetic mechanisms silence or promote gene expression and allow cells to fine-tune their responses to physiological stimuli (8). While research in the past few decades focused on DNA methylation and histone modifications (9, 10), the focus shifted more recently toward understanding the role of miRNAs in regulating cellular machinery, extracellular remodeling, and cytokine synthesis (11).

Initially assumed to regulate only the functions of their cell of origin, miRNAs were later found to be secreted into the extracellular fluid and regulate other target cells, thereby suggesting their role in intercellular communication (12). Like protein-coding genes, the genes encoding miRNA are located throughout the genome and commonly exhibit sequence conservation among vertebrates. They can be classified into several categories as described by Liu et al. (13); however, the two major categories (*intergenic* and *intragenic*) are well described in the literature. *Intergenic* miRNA genes are independent genes with their promoters, while *intragenic* genes are processed from introns and exons of protein-coding genes (14). Most intragenic miRNAs share common regulatory mechanisms and expression patterns with the host gene (15, 16). This implies that miRNA biogenesis is a multistep synthesis process, with each step localized in different subcellular compartments making it a tightly regulated mechanism (17).

**Figure 1:**
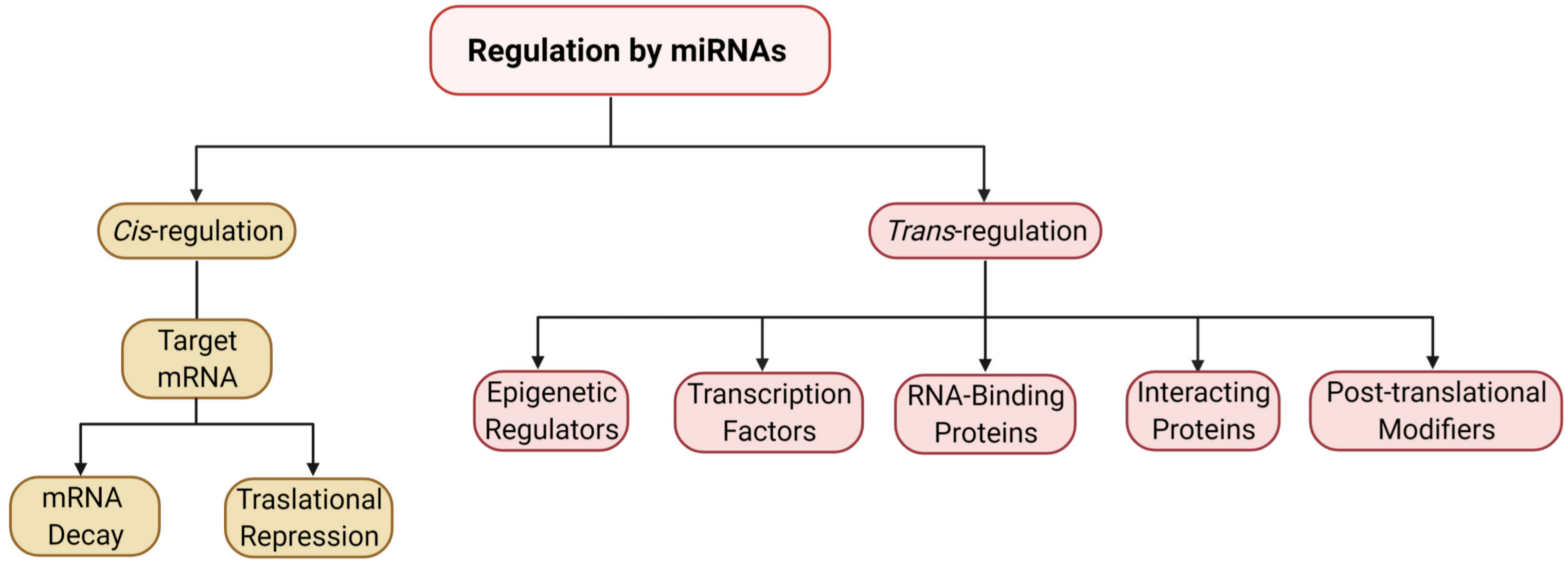
miRNA-mediated regulation of protein expression. The flow chart represents the mechanisms of gene expression modulation by miRNAs, both *cis*- and *trans*-regulatory pathways.

miRNAs regulate protein translation from target mRNA in a *cis* or *trans* manner. In *cis*-regulation, miRNAs partner with Argonaute (Ago) proteins to form a miRNA-induced silencing complex (miRISC) that either blocks translation or induces mRNA cleavage upon binding with miRNA recognition element (MRE) within the 3’ untranslated regions (UTRs) of target mRNA (2). In *trans* regulation, a miRNA represses protein translation by coordinating with key transcription factors, initiation factors, RNA-binding proteins, or enzymes to disrupt the 43S preinitiation complex or post-translational modifiers (**Figure 1**) (18–21).

## miRNA in rheumatic diseases: what we know and what we don’t

**Figure 2:**
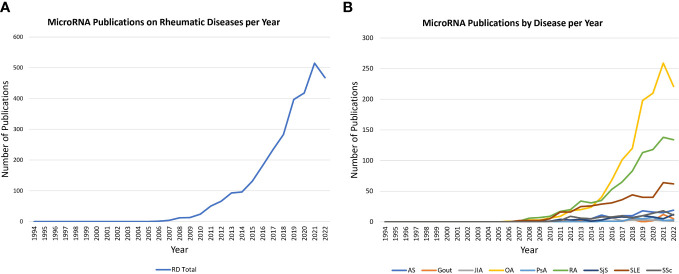
Trends in miRNA publications on PubMed. **(A)** Line graph illustrates the rise in miRNA publications on rheumatic diseases (total; 1994 to 2022). The data reveals a marked increase in publication numbers annually since 2005. **(B)** Line graph shows trends in miRNA publications across nine rheumatic diseases under study (1994-2022). The highest increase is shown for OA, RA and SLE, highlighting a research opportunity to further investigate the potential role of miRNA in the other six diseases.

The number of primary research publications on miRNAs and their role in rheumatic disease conditions has increased dramatically in recent decades, as shown in **Figure 2A**. While the numbers are growing, published studies in rheumatic diseases have primarily focused on either the functions of a miRNA regulating several genes in the pathogenesis of a disease or broad identification of miRNAs dysregulated in a specific rheumatic disease or related cell types. As a result, despite marked progress in understanding key miRNA in cancer biology and cardiovascular diseases that has led to the development of miRNA-targeted therapies, our understanding of potential therapeutic target miRNA(s) in common rheumatic diseases remains elusive (**Figure 2B**). Therefore, it is imperative to give meaningful direction to future studies on miRNA in rheumatic diseases to narrow the widening gap compared to the progress in other chronic diseases. With that objective in mind, we performed this computational algorithm-based literature search on miRNAs in rheumatic diseases to help identify key common miRNAs that are a driving force in disease processes to facilitate targeted therapeutic approaches initiated as treatment options. This method of current literature analysis also identified miRNA(s) that are uniquely implicated in specific rheumatic diseases.

More than 100 rheumatic diseases afflict humankind, and millions of US citizens are affected. Rheumatic diseases usually affect joints, tendons, ligaments, bones, and muscles. Importantly, some are autoimmune inflammatory conditions, while others (e.g., OA and gout) have different etiology. Rheumatic diseases are considerable health and socioeconomic concerns as they progress over time, causing disability, severe co-morbidities, or early mortality. Studies have shown that genetic predisposition, environmental stimuli, and epigenetic factors contribute to the onset and progress of rheumatic diseases, with increasing evidence suggesting that miRNAs have a substantial functional impact on disease progression (22, 23). Dysregulated expression of miRNAs has been reported in rheumatic diseases like rheumatoid arthritis (RA), systemic erythematosus lupus (SLE), gout, juvenile idiopathic arthritis (JIA), Sjogren’s syndrome, osteoarthritis (OA), scleroderma, psoriasis, and others. The regulation of miRNAs is affected by various proteins, often involving protein-protein and protein-RNA interactions (24). Knowledge of these processes has provided insights into the disease-specific dysregulation of miRNAs and the subsequent impact on disease pathogenesis. In this review, we utilized a systematic approach of combing the available literature to shortlist the topmost influential miRNAs in rheumatic diseases to decipher their role in disease pathogenesis. Furthermore, we discuss factors in favor of or against targeting them for therapeutic interventions.

## Computer application-guided literature review strategy

Our review covers miRNA involvement in nine prevalent rheumatic diseases, some auto-immune inflammatory conditions, and some with other disease etiology: ankylosing spondylitis, gout, JIA, OA, RA, psoriatic arthritis, Sjogren’s syndrome, SLE, and systemic sclerosis. To create a comprehensive list of miRNAs associated with rheumatic diseases, we searched PubMed for primary research publications through August 29^th^, 2023, that included the terms “microRNA” or “miRNA” or “miR” and one rheumatic disease at a time, e.g., ((((miR[Title/Abstract]) OR (miRNA[Title/Abstract])) OR (microRNA[Title/Abstract])) AND (Disease name [Title/Abstract])) NOT (Review[Publication Type]). Using the *save citations* to *file* menu option, we saved *all results* in *PubMed* format. This search captured the following number of primary publications per disease: ankylosing spondylitis (113), gout (32), JIA (21), OA (1277), psoriatic arthritis (24), RA (822), Sjogren’s syndrome (74), SLE (417), and systemic sclerosis (103). A total of 2,883 articles divided into nine files (one per disease) were sorted for their relevance to a specific rheumatic disease based on their currently published primary articles using Windows PowerShell scripting language. The Windows PowerShell Integrated Scripting Environment (ISE) is an in-built application of Windows also supported by Linux and macOS products at no additional cost. It can be learned conveniently through manuals and online resources. All nine search files downloaded from PubMed were read in PowerShell to locate the term “miR” followed by a number in the title or abstract of all the articles. Appropriate coding ensured that none of the unique miRNAs were counted more than once per article. The resulting output file for each rheumatic disease contained a comprehensive list of all the miRNAs found in primary research publications for that disease, with a total for each miRNA.

**Figure 3:**
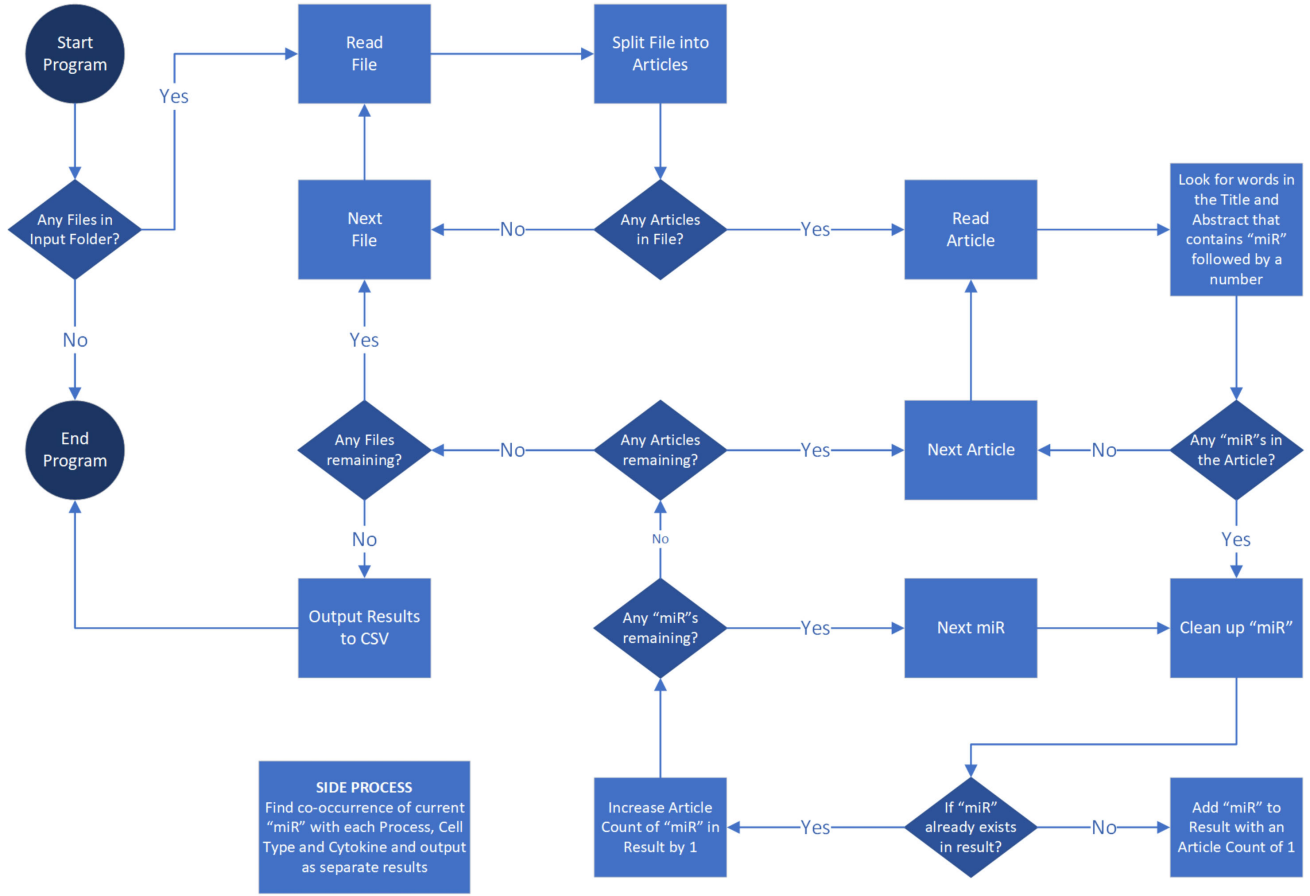
Literature review strategy employing PowerShell algorithm. The flow chart outlines the coding process for systematically screening primary research publications on PubMed.

Individual miRNAs can positively or negatively affect disease pathogenesis through their dysregulated expression in certain cell types, effect on specific biological processes, or regulation of cytokines. For example, our laboratory has reported that miR-17 negatively regulates TNF-α inflammatory signaling but that its expression is unusually low in rheumatoid arthritis synovial fibroblasts (RASFs) (20). To understand the significance of miRNAs to each disease, we compiled a list of pathological processes (angiogenesis, apoptosis, autoantibody, biomarker, degeneration, diagnosis, fatigue, fibrosis, inflammation, invasion, metabolism, migration, pain, proliferation, and therapy), cytokines (GM-CSF, IL-1, IL-2, IL-6, IL-8, IL-18, IL-17, IL-10, TNF, RANKL and interleukin), and cell-types (macrophage, dendritic cells, monocyte, T-cell, T-reg, B-Cell, fibroblast, osteoclast, osteoblast, endothelial, epithelial, chondrocyte, nerve and neuron). The abstract and the title of all the articles were further mined for the co-occurrences of a unique miRNA and each of the mentioned processes, cytokines, or cell types to count (once per article) the unique co-occurrence of each combination. See the flow chart in **Figure 3** for the graphic depiction of the coding steps.

## Three most published miRNAs in rheumatic diseases: miR-146, miR-155 and miR-21

**Figure 4:**
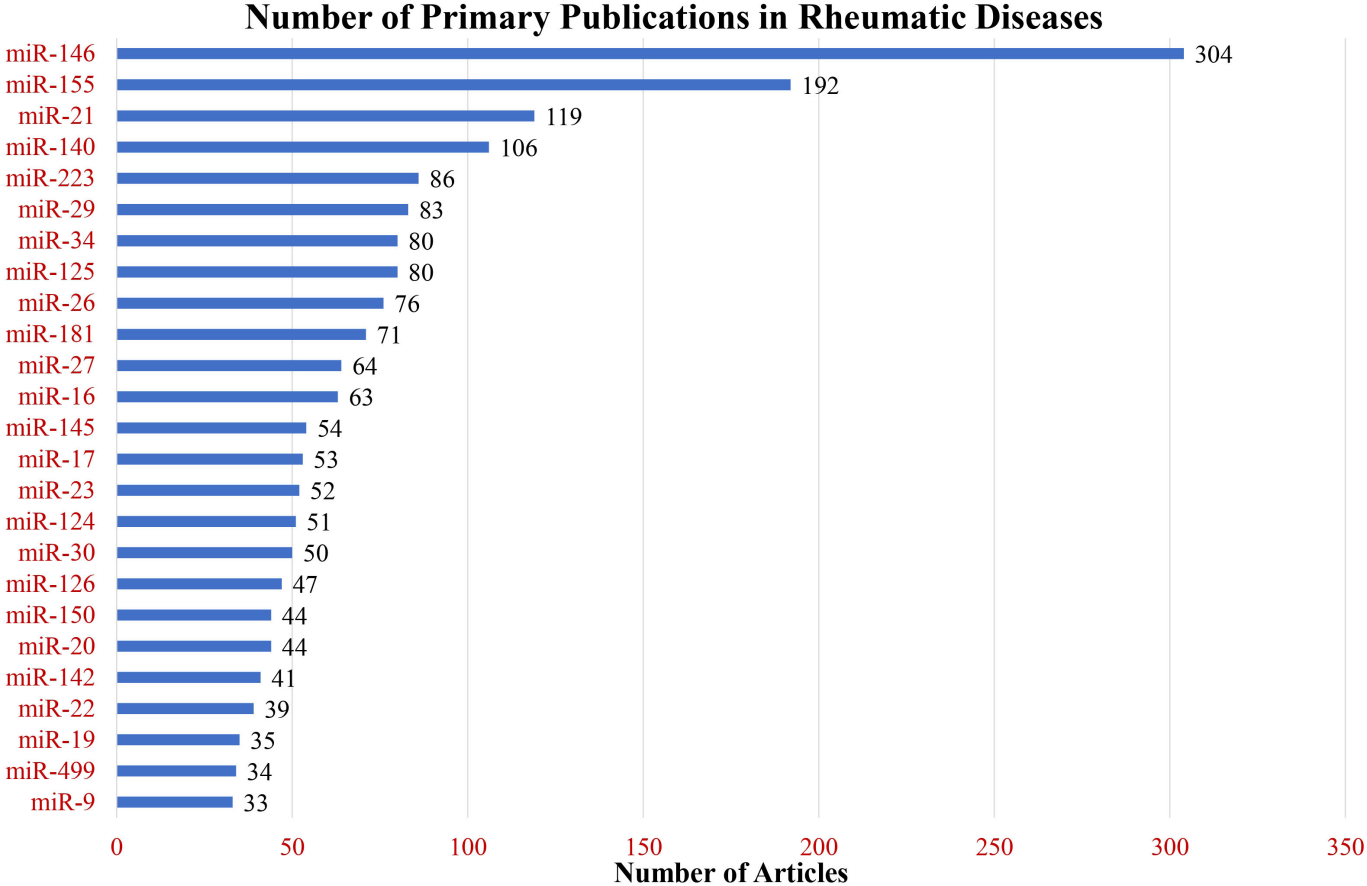
Primary research publications in rheumatic diseases. The bar graph illustrates the number of publications for the top 25 most-published miRNAs across nine rheumatic diseases under study.

**Figure 5:**
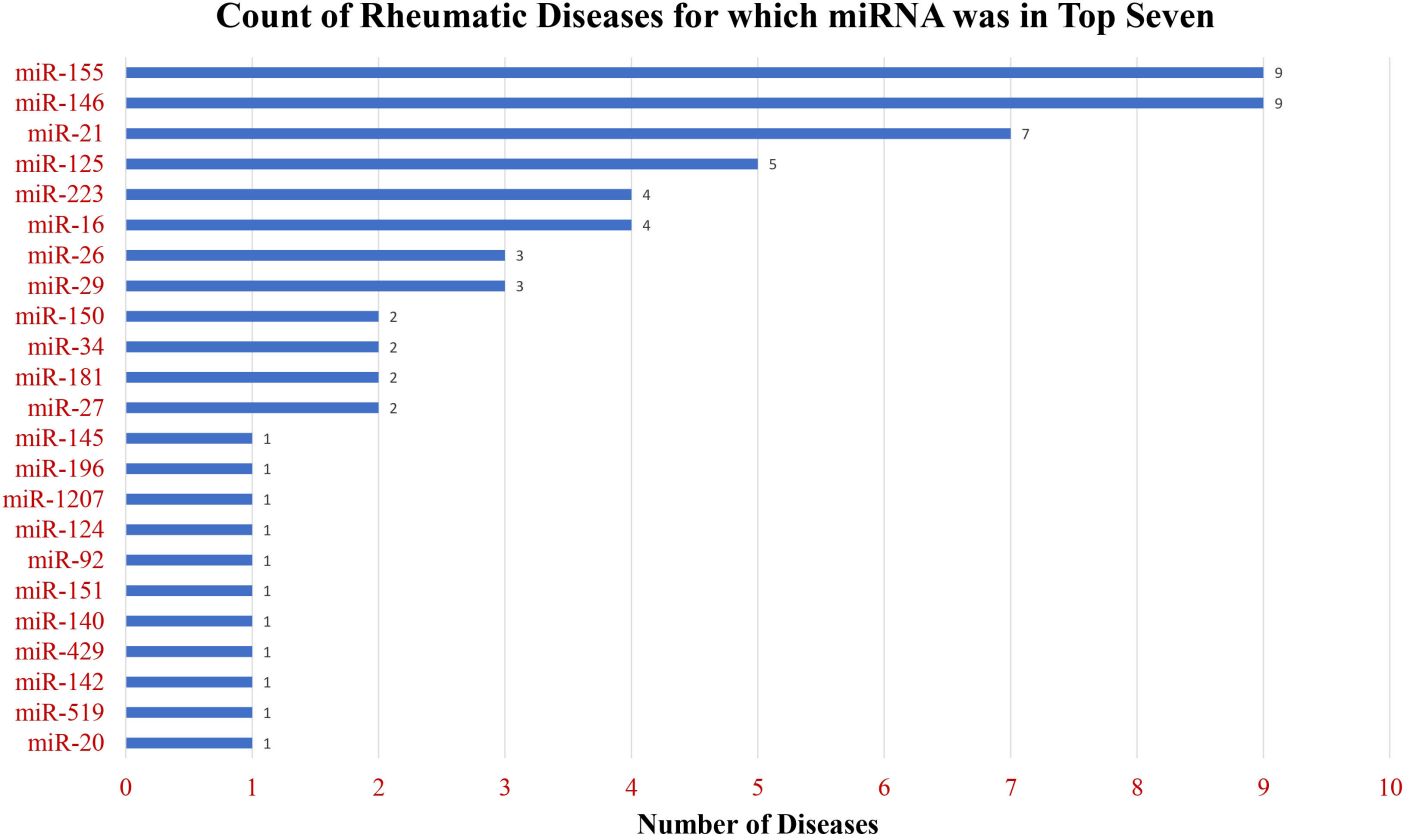
Breadth of publications across diverse rheumatic diseases. The chart displays the count of rheumatic diseases for which miRNAs ranked in the top seven among the most frequently mentioned miRNAs, showcasing the widespread relevance of these miRNAs across various rheumatic conditions.

The total number of primary research articles in PubMed referring to individual miRNA and nine common rheumatic diseases are depicted in **Figure 4**. The top three miRNAs based on overall count were miR-146, miR-155, and miR-21. Interestingly, these were also the top 3 miRNAs based on the number of rheumatic diseases in which they were in the top seven (**Figure 5**). These top three most-published miRNAs are described in the following sections.

### miR-146

miR-146 is the most-published miRNA in rheumatic diseases except gout and systemic sclerosis. Mention of miR-146 frequently co-occurred with the cell types of fibroblasts or monocyte/macrophages. The title or abstracts mentioning miR-146 most frequently referenced inflammation as well as inflammatory cytokines IL-1, TNF, IL-6, and IL-17. The research focus on miR-146 was unique in OA, where it was associated with chondrocytes and their functions, including proliferation and apoptosis.

miR-146 family includes miR-146a and miR-146b, which are encoded on chromosomes 5 and 10, respectively. Following a groundbreaking publication in 2006 by Taganov et al., miR-146a and miR-146b have been highly studied in the context of innate immunity and inflammation (25). The authors demonstrated that miR-146a and miR-146b are induced in human THP-1-derived macrophages by inflammatory stimuli, including bacterial lipopolysaccharide, TNF-α, and IL-1β through the NF-κB signaling pathway. They were also the first to propose that miR-146a and miR-146b function as negative-feedback regulators of cytokine and toll-like receptor 4 (TLR-4) pathways by targeting the transcripts of key cytokine signaling intermediates such as IL-1 receptor-associated kinase 1 (IRAK1) and TNF receptor-associated factor 6 (TRAF6). Although miR-146a and miR-146b share the same seed sequence, the influence of shared and unique transcription factors they engage leads to their different temporal and tissue-specific expression (26). For example, comparing the effects of various inflammatory cytokines on human retinal epithelial cells, Kutty et al. reported maximal upregulation of miR-146a with IL-1β stimulation, while maximal miR-146b expression was observed with IFN-γ (27).

In both healthy individuals and RA patients, the expression of miR-146 and miR-155 exhibits a high degree of correlation, yet their biological effects are opposite. Increased expression of miR-155 and miR-146a has been reported in synovial tissues, lymphocytes, peripheral blood-derived mononuclear cells (PBMC), and whole blood samples of RA patients compared to healthy individuals (28, 29). While the upregulation of miR-146a has protective roles, miR-155 is implicated in the pathogenesis of rheumatic diseases. A study by Mann et al. demonstrated cross-regulation of miR-146 and miR-155, where unchecked expression of miR-155 led to chronic inflammation in miR-146-deficient mice. Their results suggest that miR-146a and miR-155 fine-tune NF-κB activity to regulate macrophage inflammatory responses (30–32).

miR-146a is a negative regulator of inflammatory signaling in RA, gout, and SLE. It targets several autoantibody-induced TLR signaling components through IRAK-1 and TRAF6 and reduces the uncontrolled production of type I IFN (33). Individuals with autoimmune diseases such as RA or SLE are at greater risk for comorbidities such as cardiovascular disease and renal damage due to the chronic burden of systemic inflammation (34–36).

The highly conserved Notch signaling pathway plays a pivotal role in the polarization of macrophages toward the pro-inflammatory M1 phenotype. Huang et al. demonstrated that miR-146a targets and suppresses Notch1 activity, reducing M1 macrophage differentiation and facilitating polarization toward the anti-inflammatory M2 phenotype. Peroxisome proliferator-activated receptor γ (PPARγ), induced in IL-4-stimulated macrophages, is a marker and promoter of M2 macrophage activation with known anti-inflammatory effects. Through the use of an inhibitor and mimic of miR-146a, Huang et al. showed that, in addition to its inhibitory impact on Notch1, miR-146a contributes to the M2 macrophage phenotype by activating PPARγ, emphasizing its involvement in macrophage polarization (37).

The miR-146 family is crucial in preventing dysregulated humoral immune responses and production of autoantibodies. Cho et al. demonstrated that miR-146a and 146b function in a cell-specific manner to regulate the germinal center (GC) reaction between GC B cells and follicular T helper cells, acting as molecular brakes during this critical precursor step to antibody production (38). The authors found that a mouse model with B cell knockout of miR-146a showed increased amounts of IKKα and c-Rel proteins in GC B cells, and a luciferase reporter study confirmed that miR-146a can directly repress these two targets in the CD40 signaling pathway that leads to GC B cell differentiation. In that same study, a mouse model with T cell knockout of both miR-146a and its paralog miR-146b showed spontaneous accumulation of follicular T helper cells and GC B cells, with higher levels of experimentally induced antibody production.

Overall, miR-146 plays an important role in the downregulation of inflammation and tissue injury in common rheumatic diseases with inflammatory pathogenesis. Whether the preventative approaches designed in the early stages of disease pathogenesis through exogenous delivery of miR-146 can retard disease progression remains to be tested.

### miR-155

miR-155 is the second most studied miRNA in rheumatic conditions, based on the number of primary research publications in our PubMed search. Its breadth of importance is notable by its appearance in the top seven miRNAs for all the nine diseases selected here for review. As mentioned earlier, miR-155 and miR-146 demonstrate co-expression. This was reflected in their similar publication pattern, with frequent co-occurrence in abstracts describing fibroblasts, monocyte/macrophages, inflammatory cytokines IL-1, IL-6, IL-17, TNF, and the process of inflammation. Like miR-146 and other miRNAs, miR-155 was most often referenced with the cell-type chondrocytes in OA. Independently, miR-155 in some studies was linked with processes involved in cellular fate, such as apoptosis and proliferation, fibrosis in systemic sclerosis, and autoantibody in SLE.

This miRNA is widely studied for its pro-inflammatory activities and its upregulation in autoimmune diseases. miR-155 has been shown to have indispensable roles in the regulation of immune cells like T-cells, B-cells, and dendritic cells (39). miR-155 has been shown to directly target and suppress the negative regulator of T cell activation, cytotoxic T lymphocyte-associated antigen (CTLA-4), with overexpression of miR-155 resulting in proliferative responses of helper T cells (40). Notably, CTLA-4 has lower expression in regulatory T cells (Tregs) from RA patients compared to healthy individuals (41). With their anti-inflammatory roles, Tregs are widely studied to be potentially therapeutic in autoimmune diseases. It has been shown that Tregs require the cell surface antigen CTLA-4 for their suppressive action in inflammation (41).

In the context of autoimmunity, upregulation of miR-155 is deleterious as it can amplify inflammation by promoting Th17 helper T cell differentiation, B cell maturation and autoantibody production, T cell-dependent inflammation, and dendritic cell production of TNF-α, IL-6, and IL-17 (42). Kurowska-Stolarska et al. discovered that miR-155 also promotes inflammation by targeting an inhibitor of inflammation, Src homology 2-containing inositol phosphatase-1 (SHIP-1), in RASFs (43). They found that miR-155-deficient mice failed to develop collagen-induced arthritis (CIA), attributing this to the indispensable role of miR-155 in the development of cytokine-producing Th17 cells and the activation of B cells necessary for autoantibody production. To date, most studies have established miR-155 as a positive regulator of inflammation in RA and other rheumatic diseases. Stypinskac et al. reported an 8.6- and 3-fold increase in circulating miR-155 in SLE and systemic sclerosis, respectively (44).

The association of high levels of miR-155 with reduced levels of the suppressor of cytokine signaling, SOCS1, has been reported in inflammatory conditions such as severe acute pancreatitis, ulcerative colitis, and breast cancer (45–47). In the context of RA, Li et al. discovered that miR-155 targets the 3’UTR of the SOCS 1 mRNA and suppresses its expression in RA peripheral blood and peripheral blood macrophages. In addition, the authors also highlighted the role of miR-155 in higher expression of TNF-α and IL-1β, where the inhibition of miR-155 significantly reduced the production of these cytokines in the supernatant of PBMCs after 24 hours of LPS treatment (48). In another study, Kmiolek et al. looked at transcription factors critical in maintaining Th17 and Treg balance: SOCS1, SMAD3, SMAD4, STAT3, STAT5, to study their correlation with miR-155 and other select miRNAs in RA, OA, and healthy subjects. Their findings show correlations between miR-155 and STAT3 in Th17 cells in RA and between miR-155 and SMAD3 and SMAD 4 in Treg cells in RA. Notably, they also observed a strong positive correlation between miR-155 and SOCS1 in the Tregs of OA patients (49). The role of miRNA in regulating Th17/Treg balance by directly targeting transcription factors is an example of *trans* regulation of gene expression, as illustrated in **Figure 1**. Alone or in conjunction with miR-21, miR-155 promotes vasculopathy and tissue fibrosis. These processes are central to the pathology of systemic sclerosis (50). It has been shown that miR-155 mediates TGF-β1 signaling, which drives collagen synthesis in fibrosis (51). Experimental inhibition of miR-155-5p in mice reduced macrophage and fibroblast activation and attenuated silica-induced lung fibrosis (52) and valve damage in rheumatic heart disease (53). miR-155 may represent a therapeutic target to safely control fibrosis in systemic sclerosis. This is clinically relevant as the initial human trial of an anti-TGF-β drug had to be halted due to unexpected morbidity and mortality in the treatment groups (54), and despite subsequent trials of new candidates, there are still no approved TGF-β inhibitors.

### miR-21

The pattern of publications for miR-21 revealed by our text mining search is in line with a recent systematic review and meta-analysis of miRNAs in autoimmunity, which highlighted miR-21 among top miRNAs with significant differential expression across multiple autoimmune diseases (55). We found miR-21 to be the third most studied miRNA, with 119 mentions in primary research abstracts related to rheumatic conditions. It was in the top seven miRNA for all our selected diseases except ankylosing spondylitis, gout, and OA. Compared to other miRNAs, miR-21 appeared in the second-highest number of research publications in psoriatic arthritis and systemic sclerosis. In RA, miR-21 was studied in conjunction with fibroblasts, T cells, osteoblasts, and osteoclasts, with a focus on the cytokine IL-6. In systemic sclerosis, miR-21 was found in publications on fibroblasts, endothelial cells, and the process of fibrosis. In SLE, miR-21 was studied with autoantibody production.

As mentioned in the miR-155 section, miR-21 is strongly pro-fibrotic. A current PubMed search not related to rheumatic diseases reveals hundreds of publications focused on miR-21 in cardiac, renal, hepatic, or pulmonary fibrosis. miR-21 is upregulated, and miR-29a is downregulated in dermal fibroblasts from systemic sclerosis patients compared to non-diseased controls; furthermore, miR-21 is upregulated when dermal fibroblasts are treated with TGF-β, and TGF-β and miR-21 synergistically induce collagen production (56). Two separate reports have linked elevated levels of miR-21 in plasma and urine exosomes of SLE patients with renal fibrosis due to lupus nephritis (57, 58). A key collaborative study revealed that in addition to its known pro-fibrotic effects on cardiac fibroblasts, miR-21 contributes to fibrogenesis through effects on megakaryocytes, platelets, and their soluble products (59). In plasma samples from the community-based Bruneck Study (n=660), they found a strong correlation between miR-21 and the latency-associated peptide of TGF-β1, platelet-derived growth factor (PDGF), proplatelet basic protein (PPBP), and platelet factor 4 (PF4). The authors showed that miR-21 null mice have significantly reduced numbers of circulating platelets. Their results demonstrated that miR-21 targets Wiscott-Aldrich syndrome protein (WASp), a negative regulator of platelet activation and TGF-β production. Pharmacologic inhibition of miR-21 de-repressed WASp and significantly decreased platelet release of TGF-β, generally regarded as the “master switch” of fibrosis.

Wu et al. treated MH7A immortalized RASFs with LPS, an *in vitro* model of RA, and demonstrated the upregulation of miR-21 and cytokines IL-6 and IL-1β. LPS-induced levels of miR-21 target proteins NF-κB p65, IκB-α, PTEN, PI3K, and Akt and affected the NF-κB, PTEN, and PI3K/AKT pathways. miR-21 exerts its role by negatively regulating a cell proliferation protein suppressor, SNF5. Exogenous addition of SNF5, a transcription regulatory protein, reduced p-NF-κBp65, p-IκB-α, p-PI3K, and p-Akt proteins, thereby suppressing the inflammatory response (60).

Existing studies confirm that miR-21 plays pathogenic and protective roles in an array of conditions related to bone health. In a pivotal 2011 publication by Sugatani et al., miR-21 was shown to mediate receptor activator of nuclear factor κB ligand (RANKL)-induced osteoclastogenesis in mouse bone marrow-derived monocyte/macrophage precursors (61). In a separate study, miR-21 was identified among seven miRNAs significantly upregulated in the serum of postmenopausal women with low bone mineral density and vertebral fractures (62).

While there are currently no clinical trials for miR-21 antagonists in rheumatic diseases, the safety, pharmacodynamics, and pharmacokinetics of RG-012 miR-21 inhibitor were recently tested for the prevention of kidney fibrosis in Alport’s syndrome (NCT03373786). Of note, RA patients are being recruited for a clinical trial to track the potential effects of the Jak inhibitor tofacitinib on pain sensitization, and as a secondary outcome, investigators will be monitoring plasma levels of four miRNAs implicated in Jak/STAT signaling and RA pathogenesis, including miR-21 (NCT03815578).

## Disease-specific role of identified miRs: impact on cell types and functions

**Figure 6:**
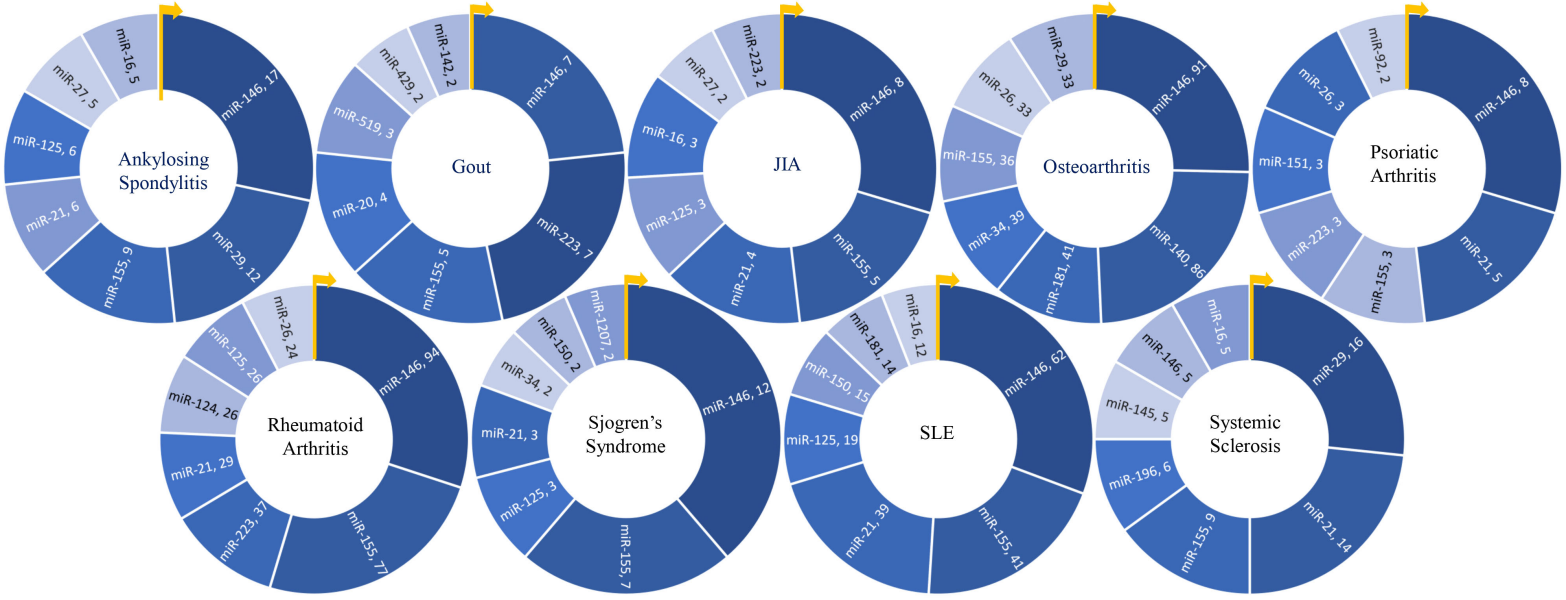
Key miRNAs studied in various rheumatic diseases. Donut charts depict the top seven miRNAs researched in each of the nine rheumatic diseases, providing an overview of the predominant miRNA research focus within each disease.

**Figure 7:**
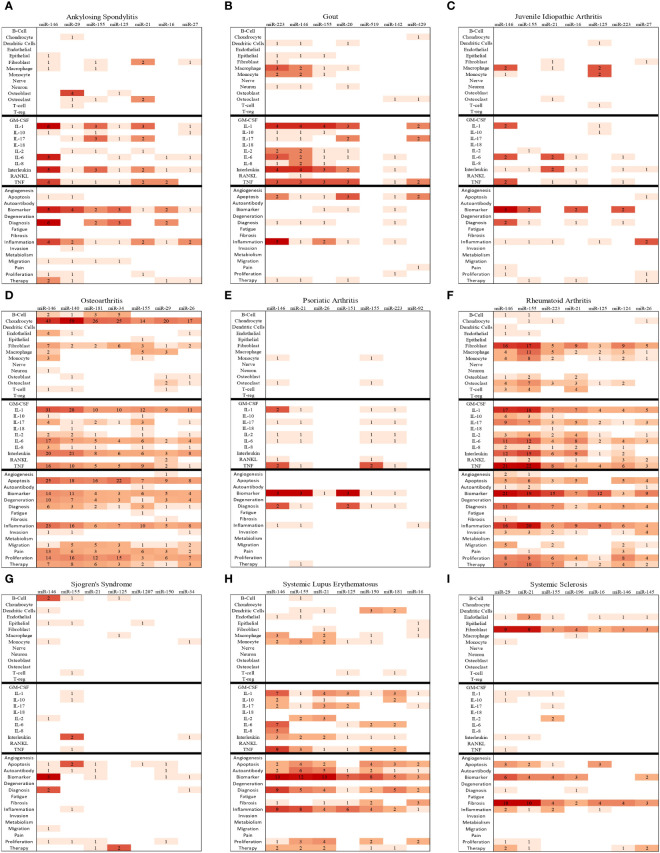
Bibliographic Heatmaps **(A–I)**: In-depth analysis of top seven miRNAs in nine rheumatic diseases. The heatmaps provide insights into the top seven miRNAs studied in the context of nine rheumatic diseases by illustrating the frequency of co-occurrences of key cell types, cytokines, and pathological processes with miRNA in the available primary publications on PubMed.

In summary of the role of top miRNA in most common rheumatic diseases, the top seven most frequently studied miRNAs for each disease are represented in **Figure 6**. Additionally, disease-specific heat maps depicting the top seven miRNAs specific to individual rheumatic conditions are shown in **Figure 7**, with color coding to indicate the frequency of publication with certain pathogenic processes, cell types, and cytokines.

## Ankylosing spondylitis

In ankylosing spondylitis (AS), miR-146, miR-29, and miR-155 were identified as key miRNAs based on our computer-guided literature mining. miR-146 was frequently mentioned in the context of cytokines like IL-1β, IL-6, and TNF-α and the process of inflammation. miR-29 appeared in four primary research articles referring to osteoblasts, whereas miR-155 appeared in three, referring to IL-1β and IL-17 cytokines. The subject of miRNAs in ankylosing spondylitis was systematically reviewed by Li et al. in 2016, and miR-146 and miR-29 were among those discussed in that review (63). miR-146a has been shown to repress the expression of the target genes of NF-κB like IL-1β, IL-6, TNF-α, and IL-8 in AS (64). In recent work, significantly reduced miR-146 expression was found in the subset of ankylosing spondylitis patients with metabolic syndrome, which was further associated with increased mRNA levels of transcription factors NF-κB and AP-1 and secreted cytokines (MCP-1/CCL2, MIP-1α/CCL3, IL-8/CXCL8 and IL-1β) (64). Polymorphisms in the miR-146 gene and the 3’UTR of miR-146 inflammatory target genes like IRAK-1 are highly studied and found to be significant in AS (65–67). miR-29 was identified as the second-most published miRNA in AS. miR-29 levels are elevated in PBMCs and osteoblasts in AS and have been shown to promote the proliferation, invasion, and migration of osteoblasts (68, 69).

miR-155 and miR-146a together demonstrate significant upregulation in AS patients, with increased miR-155 levels positively correlating with disease activity (70). Chen et al. reported that miR-155 was one of the key miRNAs upregulated in Th17 cells of AS patients (71). Th17 cell frequency is higher in AS patients, and multiple lines of evidence implicate these cells in AS pathogenesis (72, 73). Interestingly, treatment with nano-curcumin decreased miR-155 expression levels and improved clinical symptoms in AS patients (74).

## Osteoarthritis

Osteoarthritis (OA) is by far the most studied rheumatic condition with regard to the role of miRNAs, with a total of 1277 articles revealed in our PubMed search. Chondrocytes were the most extensively studied cell type in OA, with 43 abstracts mentioning chondrocytes and miR-146 and 59 abstracts mentioning chondrocytes with miR-140. IL-1, IL-6, and TNF-α were the most frequently studied cytokines in the context of OA and miRNAs: miR-146 co-occurred with IL-1, IL-6, and TNF-α in 31, 17, and 16 abstracts, respectively. miR-140 had the second-highest number of publications in OA and co-occurred with IL-1β in 28 abstracts and TNF-α in 10 abstracts.

The most frequently studied processes in OA were apoptosis, proliferation, inflammation, and degeneration. Apoptosis was mentioned in 105 abstracts with the top seven miRNAs, specifically with miR-146 in 25 abstracts and miR-34 in 22 abstracts. Proliferation and inflammation co-occurred 73 and 75 times, respectively, with the top seven miRNAs. Uniquely, the top seven miRNAs studied in OA also co-occurred more frequently with the term “degeneration” in OA than in other rheumatic diseases, often referring to degeneration of articular cartilage.

The prominence of miR-140 in OA research is not surprising, given that the expression of this anti-arthritic miRNA is almost exclusive to chondrocytes (75). It is encoded in an intron within the WW domain-containing protein 2 (Wwp2) gene locus (76). miR-140 plays key roles in bone growth and cartilage homeostasis. miR-140 (-/-) mice show skeletal growth retardation and cartilage fibrillation, while transgenic mice with cartilage-specific miR-140 overexpression have higher proteoglycan and collagen levels and show resistance to antigen-induced arthritis (77). These effects are attributed in part to miR-140 targeting and suppressing the mRNA of ADAMTS5, a proteolytic enzyme implicated in articular cartilage degradation (77). miR-140 has also been shown to target DNPEP, an antagonist of bone morphogenic protein signaling, which is critical for endochondral bone formation (75).

Levels of miR-140 are tuned through interactions with other non-coding RNA. Circular RNA S-phase cyclin A associated protein in the endoplasmic reticulum (circSCAPER) is highly expressed in IL-1β-treated chondrocytes and OA cartilage and has been reported to act as a “sponge” of miR-140-3p (78). miR-140 was also shown to be the target of long non-coding RNAs LINC01385 (79) and plasmacytoma variant translocation 1 (PVT1) (80). Restoration of miR-140 is being tested as a therapeutic approach in preclinical models of OA. Exosomes from synovial mesenchymal stem cells overexpressing miR-140-5p (SMSC-140-Exos) have been shown to stimulate the proliferation and migration of articular chondrocytes *in vitro* (81). The same authors reported that SMSC-140-Exos successfully prevented OA in a pre-clinical rat model.

Several studies have reported increased expression of miR-146 in the clinical samples of OA patients (82–84). Shao et al. reported miR-146 as one of the top differentially expressed miRNAs between healthy control cartilage and OA cartilage. Furthermore, their results suggested that miR-146 modulates IL-1β-induced chondrocyte apoptosis via the TRAF-6/NFκB pathway (83). Paradowska-Gorycka et al, reported a positive correlation between circulating miR-146 in serum and RORc transcription factor mRNA in the whole blood of OA patients (85). RORc is the primary transcription factor that regulates Th17 cell development and makes them resistant to apoptosis, and its elevated expression is reported in several arthritic diseases (86).

miR-34 was frequently described in OA research compared to other rheumatic diseases in our review. miR-34a-5p has reported involvement in cellular processes that are central to the pathogenesis of OA, such as IL-1β-mediated inflammation and chondrocyte apoptosis (87). Abouheif et al. showed that silencing miR-34a markedly inhibited IL-1β-induced apoptosis of rat chondrocytes *in vitro* (88). The findings of Zhang et al. suggested that miR-34a contributes to OA progression by promoting chondrocyte apoptosis by targeting DLL1 and modulating the PI3K/AKT pathway (89). Tian et al. found miR-34a-5p to be up-regulated in OA tissues and IL-1β-treated primary human chondrocytes compared with non-diseased tissues and untreated chondrocytes (90). Their results suggested that long non-coding RNA small nucleolar RNA host gene 7 (SNHG7) acts to sponge miR-34a-5p, while miR-34a-5p targets the mRNA of synoviolin 1 (SYVN1) (90). Overexpression of SNHG7 or transfection of chondrocytes with a miR-34a-5p inhibitor promoted chondrocyte proliferation.

## Rheumatoid arthritis

Our search revealed a total of 822 primary publications in PubMed for miRNAs and rheumatoid arthritis. The top seven most-published miRNAs were miR-146, miR-155, miR-223, miR-21, miR-125, miR-124 and miR-26. Notably, miR-146 and miR-155 were the top candidates, with 94 and 77 publications, respectively. The remaining top miRNAs each appeared in 24 to 37 primary research article abstracts.

MicroRNAs are hypothesized to mediate gene-environment interactions underlying the development of autoantibodies in RA. Kurowska-Stolarska et al. reported that miR-155 deficient mice were resistant to the development of autoantibody responses in the collagen-induced arthritis animal model of RA (43). Anaparti et al. found altered miRNA expression profiles in the peripheral blood of rheumatoid arthritis patients and their asymptomatic first-degree relatives (both groups positive for anti-citrullinated peptide antibodies, ACPA+) when compared to healthy controls (HCs) [(91)]. Both ACPA+ study groups showed upregulated expression of miR-103a-3p, miR-155, miR-146a-5p, and miR-26b-3p and downregulated expression of miR-346 in contrast to HCs. Results of the study suggest that expression profiles of key miRNA may associate with autoantibody development and serve as prognostic markers of preclinical RA.

Our literature analysis showed that miR-146 has been the focus of study in four key cell types in rheumatoid arthritis: synovial fibroblasts, macrophages, monocytes, and osteoclasts. For example, the results of Liu et al. suggested that miR-146a suppresses synovial fibroblast proliferation and inflammatory mediators by inhibiting the TLR4/NF-κB pathway (92) miR-146 frequently appeared with the terms “biomarker”, “inflammation”, “proliferation”, and “therapy”. Due to its anti-inflammatory and anti-proliferative effects, miR-146 was frequently mentioned in the context of targeted delivery approaches for therapeutic purposes in RA.

Our search indicated that miR-155 is frequently studied in the context of fibroblasts, monocytes, and macrophages. For example, Paoletti et al. showed that higher expression levels of miR-155 were linked to a defect in macrophage polarization in RA, which is differentially affected by distinct TNF inhibitors (93). Only monocytes from the blood of RA patients, in contrast to monocytes from healthy donors, were resistant to experimentally induced polarization to anti-inflammatory M2-like macrophages, and this correlated with levels of miR-155 (93). Like miR-146, over a third of publications mentioned miR-155 with the terms “biomarker” and “therapy” in RA.

miR-223 and miR-21 were also frequently studied in RA, with 37 and 29 primary research articles, respectively. The role of miR-223 in immune cell differentiation and inflammation was recently reviewed by Jiao et al. (94). The effects of miR-21 are cell-type specific. For example, through miR-21 mimic and inhibitor transfection studies, Wu et al. found that miR-21 promoted LPS-induced inflammatory responses in the MH7A cell line by activating NF-κB and PTEN/PI3K/AKT pathways via silencing the tumor suppressor gene SNF5 (60). Geest et al. showed that miR-21 has higher expression in the T-reg cells with a memory phenotype from the synovial fluid of RA patients in comparison with the conventional T-reg cells isolated from the peripheral blood of RA patients. The authors suggest that due to the anti-apoptotic roles of miR-21, its differential expression can render the memory T-reg cells resistant to apoptosis, thus modulating the overall turnover rate of T-reg cells in RA (95).

miR-26 was the sixth most published miRNA in the context of RA. miR-26 frequently appeared in the same abstracts with the terms “fibroblast”, “IL-17”, “IL-1”, “biomarker”, and “invasion”. Niimoto et al. showed that miR-26 was one of the top six most upregulated miRNAs in Th17 cells expanded from CD4+ T cells (96). Kmiołek et al. performed a receiver operating characteristic (ROC)–AUC (Area Under Curve) analysis to calculate the potential value of miRNA as biomarkers in RA and OA. Their results showed higher expression of miR-26 in T-reg cells from healthy controls compared to T-reg cells from RA patients. The difference in miR-26 levels in T-reg cells showed a significant relationship to disease classification [healthy vs. RA] (AUC 0.92, p = 0.0002), as well as in Th17 cells (AUC 0.75, p = 0.02). Furthermore, they found that miR-26 expression in RA Th17 cells positively correlated with transcription factors SMAD3, STAT3, and SOCS1 (49). miR-26 has been experimentally validated to target the mRNA of the anti-hypertrophic protein- glycogen synthase kinase-3β (GSK-3β) (97), as well as mRNA of Ezh2, a suppressor of skeletal muscle cell differentiation (98).

As shown in the heatmap, miR-124 co-occurred with the term “proliferation” in eight primary research abstracts (**Figure 7**). Several studies have reported that miR-124 expression is reduced in RA. For example, Lin et al. reported that miR-124-3p is downregulated in purified CD4^+^ cells purified from PBMCs of RA patients compared to that of healthy donors, and its expression has a negative correlation with inflammatory gene Yin Yang 1 (YY1) (99). In another article, lower expression of miR-124 was found in the synovial tissue of RA patients compared to the normal synovial tissues and inversely correlated with the expression of Histone deacetylase 1 (HDAC1) known to cause synovial hyperplasia and inflammation in RA (100). In the same publication, Meng et al. revealed that miR-124 repressed the JAK/STAT signaling pathway in CIA and alleviated hyperplasia and inflammation of the synovium (100). Another study showed that miR-124 suppresses the NFATc1-mediated pathway and inhibits RANKL-dependent and -independent osteoclast differentiation (101). In a recent placebo-controlled phase II study, the efficacy and safety of ABX464, an upregulator of miR-124 biogenesis, were determined in RA patients. The authors concluded that ABX464 at 50 mg per day or less is safe and warrants further testing in the treatment of RA (102).

## Systemic Lupus Erythematosus

Our analysis showed that miR-146, miR-155, and miR-21 were the top three most published miRNAs in the context of Systemic Lupus Erythematosus (SLE). These miRNAs frequently co-occurred with the terms “biomarker”, “diagnosis”, and “inflammation”. Interestingly, the top seven miRNAs in SLE were not associated with specific cell types in primary publication abstracts, with only a few abstracts co-occurring with macrophages and monocytes.

Xu et al. recently reviewed the roles of miR-155 in inflammatory autoimmune diseases. Their systematic analysis of the literature on miR-155 in SLE suggests that miR-155 may be a potential biomarker for predicting lupus nephritis (LN), a major complication of SLE. They summarized the role of miR-155 in the induction of inflammatory downstream signaling that leads to the development of SLE (103). In a separate publication, Khoshmirsafa et al. showed that the levels of miR-155 and miR-21 were significantly higher in PBMCs from patients with active nephritis than in subjects with absent or inactive nephritis or healthy control groups. Their ROC and logistic regression analyzes showed that these miRNAs were possible biomarkers and high-risk factors with significant roles in LN pathogenesis.

Studies suggest that miR-155 is central to autoantibody development in SLE. Thai et al. demonstrated that deletion of miR-155 in the death receptor deficient (Faslpr) lupus-prone mouse resulted in reduced autoantibody responses, with lower serum IgG anti-dsDNA antibodies and reduced kidney inflammation in this animal model of SLE (104). Leiss et al. reported significantly lower anti-dsDNA, anti-chromatin, and anti-histone autoantibody levels in miR-155-deficient mice in the pristane-induced lupus preclinical model of SLE (105).

miR-21 was one of the top three miRNAs studied across the selected rheumatic diseases, which was also prevalent in primary publications on SLE. It was mentioned in the abstracts of 39 of the total SLE miRNA articles. miR-21 co-occurred most with the terms “biomarker” and “autoantibody” in SLE, with 13 and 5 co-occurrences, respectively. Significantly elevated expression of miR-21 has been consistently reported by several researchers in serum exosomes and plasma of SLE patients compared to healthy controls, suggesting its potential as a key biomarker (106–108). Interestingly, Amr et al. showed that the plasma levels of miR-21 positively correlated with the SLE disease activity index (SLEDAI) score and proteinuria in these patients (108). Gao et al. recently showed that antagomir-based inhibition of miR-21 ameliorated the disease state by inhibiting T follicular helper (Tfh) cell expansion in the MRL/lpr mouse model of SLE (109).

Like several other rheumatic diseases, miR-146 was the focus of the highest number of publications in SLE, with a total of 62. Co-occurrences of miR-146 and various cytokines were evenly distributed across the different cytokines, with TNF-α (9), IL-1 (7), and IL-6 (7) being the most frequent. In a comprehensive study by Perez-Hernandez et al., exosomal miR-146 levels were quantified in 41 patients with SLE and 27 patients with LN, compared to 20 healthy controls. Their work shows that miR-146 has a negative correlation with SLE activity and proteinuria that can discriminate LN patients from SLE patients without LN. In addition, miR-146 was found to target the mRNAs for IRAK1 and TRAF6, suppressing the inflammation mediated by these molecules (110).

## Systemic sclerosis

Systemic sclerosis (SSc) showed unique patterns in miRNA publications compared to other rheumatic diseases, with an emphasis on the cell type fibroblasts and the process of fibrosis. Our search revealed a current total of 103 primary research articles on miRNA in PubMed for this disease. The top three miRNAs were miR-29 and miR-21, with 16 and 14 articles, respectively, and miR-155, with 9 articles. miR-29 co-occurred with fibroblasts in 9 articles and with fibrosis in 10 articles. miR-21 co-occurred with fibroblasts and fibrosis in 9 and 10 articles, respectively.

SSc is characterized by chronic activation of fibroblasts, which become resistant to apoptosis, with accompanying accumulation of extracellular matrix proteins in the skin and internal organs. The miR-29 family has been identified as key regulators of fibrosis in several organ types by targeting the 3’ UTR of collagen mRNAs in fibroblasts (111). Early studies showed miRNA-29a to be under-expressed in SSc skin biopsy samples and fibroblasts compared to those of non-diseased controls (112). The same study also demonstrated the involvement of miRNA-29a in post-transcriptional negative regulation of profibrotic type I and type III collagen genes (112). Furthermore, miRNA-29a has been implicated in several studies as a pro-apoptotic factor with therapeutic potential in sclerotic disease. It was reported that dermal fibroblasts from SSc patients and TGF-β-stimulated fibroblasts showed increased expression of anti-apoptotic Bcl-2 family proteins, while transfection with a miRNA-29a mimic reduced expression of Bcl-2 and Bcl-XL and restored sensitivity of SSc dermal fibroblasts to apoptosis (113). Shimada et al. reported lower expression of the chemokine CXCL17 in skin biopsy samples from SSc patients compared to negative controls (114). Their results showed that treatment of cultured fibroblasts or localized injection of CXCL17 in a bleomycin-induced SSc mouse model increased miR-29 and matrix metalloproteinase 1 (MMP-1) expression and decreased type I collagen expression and skin fibrosis in the mouse model (114).

Given its role in fibrosis, our search identified miR-21 to be frequently studied in SSc. miR-21 and miR-29a have been shown to act as pro-fibrotic and anti-fibrotic factors with opposing effects. In primary human fibroblast cultures from patients with diffused cutaneous SSc (dcSSc) and in TGF-β-treated fibroblasts, miR-21 was upregulated, and miR-29a was downregulated compared to normal or untreated controls. Inhibition of miR-21 or overexpression of miR-29a reduced *COL1A1* gene expression and collagen production (56). Of note, miR-21 is associated not only with dermal fibrosis but also pulmonary fibrosis in SSc. Yan et al. compared expression profiles of normal lung tissue vs SSc lung tissue to construct a competing endogenous RNA (ceRNA) network as predicted by multiple online databases (115). Their model analyzed interactions between long non-coding RNA and miRNA, miRNA and mRNA, and long non-coding RNA and mRNA. Three core sub-networks were identified to be associated with SSc, including LINC01128/miR-21-5p/PTX3 (Pentraxin 3 gene) (115). Wuttge et al. compared plasma levels of 46 selected miRNAs in 85 female patients with anti-centromere antibody (ACA) positive limited cutaneous SSc (lcSSc) and found miR-21-5p to be significantly elevated in patients with the severe complication of SSc-associated pulmonary arterial hypertension (APAH) (116).

miR-155 was also a top-ranking miRNA associated with SSc in our search. Artlett et al. published a study reporting miR-155 overexpression in lung fibroblasts from SSc patients and demonstrating that miR-155 expression was requisite for NLRP3 inflammasome-mediated collagen production in cultured murine fibroblasts (117). A gene expression profiling by Dolcino et al. reported the elevated expression of miR-155 in the sera of SSc patients compared to non-diseased controls and postulated a possible link between miR-155 overexpression in SSc and susceptibility of SSc patients to certain malignancies (118). Wajda et al. compared serum miRNA expression levels between 45 SSc patients and 57 healthy controls and found higher levels of miR-155 in SSc patient sera (119). Furthermore, increased miR-155 expression was associated with an early pattern of microangiopathy on nailfold video capillaroscopy as opposed to a late pattern, suggesting that miR-155 may serve as a biomarker for vasculopathy risk in SSc (119).

## Gout

Our search revealed a moderate number of publications on miRNA in gout compared to other rheumatic diseases, with a total of 32 primary research articles. The topmost studied miRNAs in gout were miR-223, miR-146, and miR-155. By order of frequency of mention in research article abstracts, monocytes and macrophages were the top-ranked cell types, IL-1 was the top-ranked cytokine, and inflammation was the top process.

miR-223 has been negatively associated with gouty inflammation. Several studies have supported the observation that miR-223 suppresses IL-1β and TNF-α production by targeting the NLRP3 inflammasome (120–122). Bohata et al. reported a negative correlation between miR-223-3p and monocyte chemoattractant protein (MCP-1) levels and a positive correlation with C-reactive protein (CRP) levels in the plasma of gout patients (123). miR-146 is also negatively associated with gouty inflammation in preclinical models. Zhang et al. reported exacerbated paw swelling, with markedly higher TRAF6 and IRAK1 gene expression and inflammatory cytokine production in bone marrow-derived macrophages from miR-146a knockout mice compared to wild-type control mice in a monosodium-urate (MSU)-induced gouty arthritis model (124). Contrasting studies report the elevated levels of miR-155 in PBMCs of gout patients compared to healthy controls and pro-inflammatory effects via targeting SHIP-1 mRNA (125), while another study reported no significant role for miR-155 in an MSU-induced mouse model of gout (126). Hence, the role of miR-155 in gout remains elusive and warrants further study.

## Sjogren’s syndrome

Sjogren’s syndrome showed a relatively high number of publications on miRNA, with 74 total. The top seven miRNAs for Sjogren’s syndrome were miR-146, miR-155, miR-21, miR-125, miR-1207, miR-150, and miR-34. In a recent publication by Kamounah et al. the authors provided a systematic review of 65 publications that examined proteomics and miRNAs in patients with Sjogren’s syndrome. In these publications, miRNA expression analysis was performed on plasma, serum, or PMBCs. The authors reported heterogeneity in the reviewed publications on the number and type of differentially expressed miRNAs (127). In our search results, miR-125 was mentioned with the term “therapy” twice. In an interesting work by Xing et al. PBMCs from primary Sjögren syndrome patients were cultured with exosomes derived from the labial gland mesenchymal stem cells SCs (LGMSCs) of healthy individuals. It was reported that high levels of miR-125 in the LGMSCs-derived exosomes bind and inhibit the plasma cell differentiation factor-PRDM1 (PR domain zinc finger protein 1, also known as BLIMP1), thus preventing the excessive differentiation of B-cells into plasma cells, an important hallmark of Sjogren’s syndrome (128). In our search, other than the frequently appearing miR-146 and miR-155, most of the miRNAs appeared in less than 3 publications. Further investigation of these unique and understudied candidates will be needed to validate their importance in Sjogren’s syndrome.

## JIA and psoriatic arthritis

For certain diseases, our literature mining revealed a smaller number of miRNA-related primary publications. In the case of JIA, the number of articles was 21 and for psoriatic arthritis it was 24. The sparse number of articles found in our search underscores the need for more studies on the role of miRNAs in these diseases.

## Conclusion and future directions

As the number of research articles on PubMed continues to expand, our bibliometric analysis provides a tool for a data-driven approach to identify the relevant miRNAs, cell types, cytokines, and cellular processes in rheumatic disease pathogenesis. Currently, PubMed contains over 2800 primary research publications on miRNA for just the nine diseases that were the focus of this review. To data mine the thousands of articles, we share a unique programming approach to text mining using the free computer program PowerShell. This report quantifies and highlights the published findings on miRNAs with shared effects in multiple rheumatic diseases as well as unique disease-specific miRNAs. Our analysis reveals that miR-146, miR-155, and miR-21 are the most studied miRNAs across the rheumatic diseases under consideration. In contrast, miRNAs such as miR-145, miR-196, miR-140, and miR-20 were uniquely studied in the pathogenesis of specific rheumatic diseases. Similarly, miR-34 was in the top seven for only osteoarthritis and Sjogren’s syndrome, while miR-146 and miR-155 were in the top seven for all nine rheumatic diseases.

Our computational literature analysis revealed a disparity among rheumatic diseases regarding the number of miRNA-focused primary research publications in PubMed. The number of publications on miRNA in OA, RA, and SLE exceeded other diseases under study. JIA and psoriatic arthritis showed particularly low counts, totaling under 25 to date. Potential challenges to miRNA research in these diseases may include a shortage of patient samples for study or a lack of an established role of epigenetics or miRNAs in pathogenesis. Based on our literature review as a relevance finder, existing publications suggest that miR-146, miR-155, and miR-21 are key regulating miRNAs in common rheumatic diseases, which provides a rationale for investigating their roles in these understudied diseases. Unique miRNAs such as miR-151 and miR-92 that appeared in the top seven published miRNAs only for psoriatic arthritis also warrant further study. Finally, investigators could consider similarities of involved cell types, cytokines, and disease processes highlighted in the heatmaps of other diseases to identify possible miRNA candidates.

**Figure 8:**
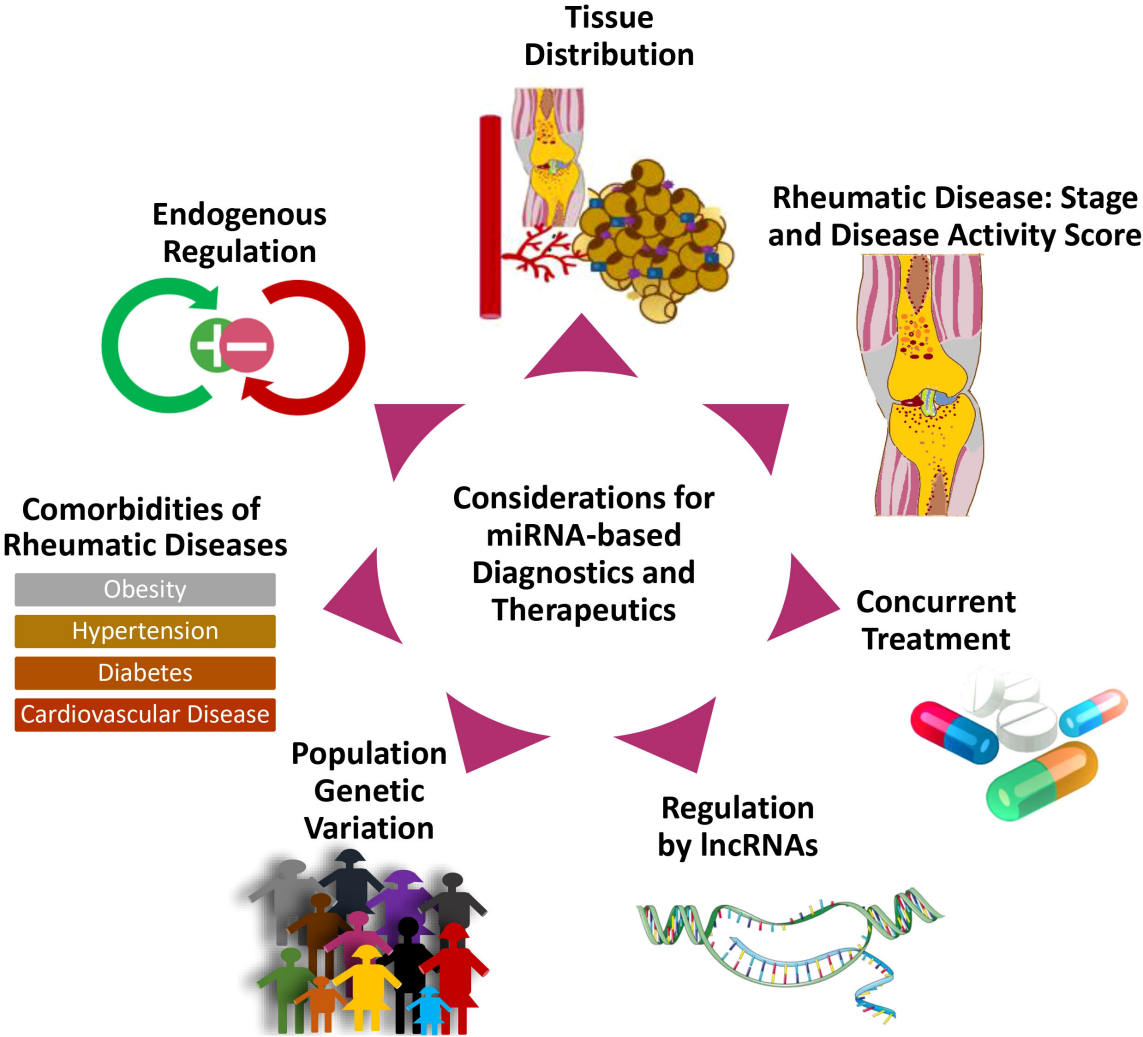
Considerations in the design of miRNA-targeted therapeutics for rheumatic diseases. The diagram illustrates considerations and challenges relevant to the development of miRNA-targeted therapies.

The authors believe that the information summarized in this review sheds light on two major categories of challenges to miRNA-based diagnostics and therapeutics (**Figure 8**). The first one includes the mechanisms of endogenous regulation, tissue-specific distribution, and the potential drug-drug interactions in miRNA-targeted therapies. The second one could be independent of miRNA status, including the epigenetic or genetic variation, disease stage or activity, associated co-morbidities, and the role of lncRNAs in rheumatic diseases (as summarized in **Figure 8**). Understanding such challenges allows researchers to consider these factors in their efforts to develop effective and safer therapies targeting miRNA in rheumatic diseases. While this review summarizes the most recent and comprehensive information on the discoveries related to the role of miRNAs in rheumatic diseases, the use of a computational approach with open-access software to serve as a relevance guide for the diverse research interests also allows precise identification of the most common and some unique disease-associated miRNAs concomitantly. Our review features preliminary evidence of the role of less explored miRNAs, reinforces the importance and interconnectedness of the topmost-studied miRNAs, and presents research opportunities in under-studied rheumatic diseases.

Our comprehensive review found no definitive information about alterations in miRNA signatures during the early versus late stages of rheumatoid arthritis (RA) and their correlation of miRNA signatures with therapeutic responses. Given the potential of certain miRNAs to emerge as biomarkers, a deeper examination of variations in these miRNAs during the disease’s early and late phases is valuable. Furthermore, there exists an opportunity to investigate whether miRNAs can predict or influence the efficacy of current therapies, emphasizing the necessity for additional research in these areas.

## Author contributions

FS: Conceptualization, Data curation, Validation, Writing – original draft, Writing – review & editing. RS: Conceptualization, Data curation, Writing – original draft, Writing – review & editing. AS: Data curation, Formal Analysis, Methodology, Software, Writing – review & editing. DF: Supervision, Writing – review & editing. SA: Conceptualization, Funding acquisition, Supervision, Writing – original draft, Writing – review & editing.
